# Genetic identification and characterization of chromosomal regions for kernel length and width increase from tetraploid wheat

**DOI:** 10.1186/s12864-021-08024-z

**Published:** 2021-09-30

**Authors:** Jieguang Zhou, Cong Li, Jianing You, Huaping Tang, Yang Mu, Qiantao Jiang, Yaxi Liu, Guoyue Chen, Jirui Wang, Pengfei Qi, Jun Ma, Yutian Gao, Ahsan Habib, Yuming Wei, Youliang Zheng, Xiujin Lan, Jian Ma

**Affiliations:** 1 State Key Laboratory of Crop Gene Exploration and Utilization in Southwest China, Triticeae Research Institute, Sichuan Agricultural University, Chengdu, 611130 China; 2 College of Agronomy and Biotechnology, China Agricultural University, Beijing, 100193 China; 3 Biotechnology and Genetic Engineering Discipline, Khulna University, Khulna, 9208 Bangladesh

**Keywords:** Tetraploid wheat, Wheat 55 K SNP array, Kernel size, QTL validation

## Abstract

**Background:**

Improvement of wheat gerc*Triticum aestivum* L.) yield could relieve global food shortages. Kernel size, as an important component of 1000-kernel weight (TKW), is always a significant consideration to improve yield for wheat breeders. Wheat related species possesses numerous elite genes that can be introduced into wheat breeding. It is thus vital to explore, identify, and introduce new genetic resources for kernel size from wheat wild relatives to increase wheat yield.

**Results:**

In the present study, quantitative trait loci (QTL) for kernel length (KL) and width (KW) were detected in a recombinant inbred line (RIL) population derived from a cross between a wild emmer accession ‘LM001’ and a Sichuan endemic tetraploid wheat ‘Ailanmai’ using the Wheat 55 K single nucleotide polymorphism (SNP) array-based constructed linkage map and phenotype from six different environments. We identified eleven QTL for KL and KW including two major ones *QKL.sicau-AM-3B* and *QKW.sicau-AM-4B*, the positive alleles of which were from LM001 and Ailanmai, respectively. They explained 17.57 to 44.28% and 13.91 to 39.01% of the phenotypic variance, respectively. For these two major QTL, Kompetitive allele-specific PCR (KASP) markers were developed and used to successfully validate their effects in three F_3_ populations and two natural populations containing a panel of 272 Chinese wheat landraces and that of 300 Chinese wheat cultivars, respectively. *QKL.sicau-AM-3B* was located at 675.6–695.4 Mb on chromosome arm 3BL. *QKW.sicau-AM-4B* was located at 444.2–474.0 Mb on chromosome arm 4BL. Comparison with previous studies suggested that these two major QTL were likely new loci. Further analysis indicated that the positive alleles of *QKL.sicau-AM-3B* and *QKW.sicau-AM-4B* had a great additive effect increasing TKW by 6.01%. Correlation analysis between KL and other agronomic traits showed that KL was significantly correlated to spike length, length of uppermost internode, TKW, and flag leaf length. KW was also significantly correlated with TKW. Four genes, *TRIDC3BG062390*, *TRIDC3BG062400*, *TRIDC4BG037810*, and *TRIDC4BG037830*, associated with kernel development were predicted in physical intervals harboring these two major QTL on wild emmer and Chinese Spring reference genomes.

**Conclusions:**

Two stable and major QTL for KL and KW across six environments were detected and verified in three biparental populations and two natural populations. Significant relationships between kernel size and yield-related traits were identified. KASP markers tightly linked the two major QTL could contribute greatly to subsequent fine mapping. These results suggested the application potential of wheat related species in wheat genetic improvement.

**Supplementary Information:**

The online version contains supplementary material available at 10.1186/s12864-021-08024-z.

## Background

Wheat (*Triticum aestivum* L.) is one of the main food crops in the world [1]. The pace of population growth requires a stable increase of wheat yield [2]. Wheat yield is determined by three key components, including productive spike number per unit area, kernel number per spike, and 1000-kernel weight (TKW) [3]. TKW is mainly affected by kernel size including kernel length (KL), kernel width (KW), and kernel thickness [4]. Therefore, KL and KW play vital roles in wheat yield formation.

To date, quantitative trait loci (QTL) for kernel size have been detected on all of the wheat chromosomes [5]. For example, seven QTL for KW were detected on chromosomes 1A, 4D, 5A, 5B, 6D, and 7B [6]. Three stable QTL were identified in more than three environments, including two for KL and one for KW [7]. Xin et al. [8] identified two QTL for KW. Furthermore, several genes for kernel size have been isolated and cloned in wheat via a map-based cloning approach. For example, the grain-shape gene *Tasg-D1* encoding a Ser/Thr protein kinase glycogen synthase kinase3 was associated with formation of round grains in wheat [9]. *Keto-acyl thiolase 2B* (*KAT-2B*) involved in β-oxidation during JA synthesis played a role in determination of kernel weight [10].

Wheat breeding is facing the bottleneck of narrow genetic basis at present [11]. Fortunately, a large diversity of undeveloped genetic resources from wheat related species could contribute to meeting future wheat production challenges. It is feasible to identify and utilize novel QTL/genes for KL and KW from excellent germplasms of wheat and its related species [12]. For example, a major QTL (*QGD-4BL*) controlling kernel size of the upper spikelet was identified in wild emmer (*T. turgidum* ssp. *dicoccoides*) [13]. Four QTL for KL and one for KW were detected in durum wheat [14]. Okamoto et al. [15] found that *P1* had a positive effect on KL in Polish wheat (*T. turgidum* ssp. *polonicum*). *TtGRF4-A* (ortholog of rice *OsGRF4*) was associated with kernel size and kernel weight in wild emmer [16].

As the progenitor of modern tetraploid and hexaploid cultivated wheat, wild emmer has the highest nucleotide diversity across the *Triticum* taxonomic groups making it an invaluable gene pool for the genetic improvement of wheat [17]. Thus, identification of QTL/genes for KL and KW from wild emmer will facilitate progress to meet wheat production challenges in the future. In this study, we are aiming at identifying and validating major QTL for KL and KW in a recombinant inbred line (RIL) population derived from a cross between a wild emmer accession and a Sichuan endemic tetraploid wheat ‘Ailanmai’.

## Materials and methods

### Genetic populations

Four bi-parent populations developed by the single-seed descent method were used in this study. They were derived from crosses Ailanmai × LM001 (AM, 121 F_8_ RILs including parents) [18], LM001 × PI 503554 (MP, 102 F_3_ lines), Ailanmai × AS 2268 (AAs, 102 F_3_ lines), and Ailanmai × PI 193877 (API, 72 F_3_ lines). Notably, the 121 RILs of AM were previously genotyped using the Wheat 55 K SNP array [18] and used for QTL mapping in this study. The other three populations were used for validating QTL identified in this study. Ailanmai (*T. turgidum* L. 2*n* = 4*x* = 28, AABB) is a local dwarf variety from Sichuan province, and LM001 is a wild emmer accession (*T. turgidum* ssp. *dicoccoides*, 2*n* = 4*x* = 28, AABB). PI 503554 (*T. turgidum ssp. durum*) and PI 193877 (*T. turgidum* ssp. *dicoccon*) were from The U.S. National Plant Germplasm System (NPGS), and AS 2268 (*T. carthlicum* Nevski) was collected and preserved by Triticeae Research Institute of Sichuan Agricultural University. Besides, two natural populations were further used to verify the effect of the major QTL, and they were: (I) a panel of 272 Chinese wheat landraces (CWL) genotyped using the Wheat 660 K SNP array [19], and (II) a panel of 300 Chinese wheat cultivars (CWC) genotyped using the Wheat 55 K SNP array [20]. The information of two natural populations was listed in Table S1.

### Phenotypic evaluation

The phenotype of AM RIL population was measured in six different environments, including Chongzhou (103°38′E, 30°32′N) in 2017, 2018, 2019, and 2020 (2017CZ, 2018CZ, 2019CZ, and 2020CZ), Wenjiang (103°51′E, 30°43′N) in 2020 (2020WJ), and Ya’an (103°0′E, 29°58′N) in 2020 (2020YA) in China. Details of all the experiments planted were consistent with previous study [18]. Field management was according to local agricultural practices [21]. Thirty kernels in each line were scanned using Epson Expression 10,000 XL. KL and KW were evaluated using WinSEEDLE (Regent Instruments Canada Inc) based on the selected objects in image [21]. Then, the average values of each line in a single environment and the best linear unbiased prediction (BLUP) value estimated from average values from different environments were used for QTL detection and further analysis. The data of spike length (SL), effective tiller number (ETN), length of uppermost internode (UIL), TKW, and grain number per spike (GNS) were retrieved from our previous study [18]. The measurement of flag leaf length (FLL) and flag leaf width (FLW) was conducted about ten days after anthesis. The FLL (from leaf bottom to the tip) and FLW (on the widest part of the leaf) were measured on five selected plants (five typical plants per row for each line) from the main tiller of each plant [22]. The phenotypic average value of each trait in multiple environments was used to calculate BLUP value of each trait for further analysis. All the observations were made during the previous experiment [18], and presentation of data of kernel size were completed in the current study along with validation of identified QTL.

The F_2_ populations of MP, AAs, and API were grown in 2020CZ and the harvested F_3_ seeds for each plant (line) were used for phenotype. Their experiment planted and field management were consistent with the AM population. Thirty kernels in each plant were scanned using Epson Expression 10,000 XL. KL and KW were evaluated using WinSEEDLE (Regent Instruments Canada Inc) based on the selected objects in image [21]. Then, the average values of KL and KW were used for validating major QTL identified in this study. Details of environmental information of agronomic traits measurement were listed in Table S2.

The 272 CWL were planted in six different environments, including 2012YA, 2013-2015WJ, and 2014-2015CZ [19]. The average value of each accession in a single environment was used for further analysis [19].

The 300 CWC were planted in three different environments, including Beijing in 2018 and 2019, and Baoding in 2019 [20]. One hundred and twenty seeds of each accession were planted in a single row of 2 m in length with 0.7 m spacing between the rows in three environments [20]. The kernel-related traits were measured using the SC-A wheat grain appearance quality image analysis system developed by the Hangzhou Wanshen Detection Technology Co [20].

### Data analysis

SAS 8.0 (SAS Institute, Cary, NC, USA) was used to analyze the BLUP of the agronomic traits and the broad-sense heritability (*H*^*2*^) in different environments. According to the description of Smith et al. [23], the SPSS Statistic 24.0 program (IBM SPSS, Armonk, NY, USA) was used to obtain Pearson’s correlation coefficients within agronomic traits based on the BLUP values, descriptive statistical analyses, and independent sample *t*-test (*P* < 0.05). Frequency distributions of KL and KW values were plotted in the Origin 9.0 software using Gaussian distribution. The individuals of the AM RILs were divided into two groups based on the genotypes of the closest markers for each of the two major QTL, and then the differences between the two groups for the corresponding traits were analyzed. Furthermore, Excel (Microsoft Corporation, Microsoft Excel 2010, USA) was used to analyze the binary linear regression analysis.

### QTL mapping

The details of DNA extraction and 55 K SNP array analysis of AM population refer to previous work [18]. The genetic map was constructed by Mo et al. [18].

The inclusive composite interval mapping (ICIM) in IciMapping 4.1 (https://www.isbreeding.net/) was used to detect QTL, and thousand permutations test (*p* < 0.05) was used for defining QTL logarithm (base 10) of odds scores (LOD) threshold [24]. A LOD score of 2.5 was chosen as a threshold for considering significant QTL [25]. The QTL × Environment (QE) interaction effects were analyzed using IciMapping with the preset parameter: step = 1 cM, PIN = 0.001, LOD = 5.0. In the present study, QTL identified in more than three environments and expressed more than 10% of the phenotype variance explained (PVE) were defined to be major ones, and those with less than 1 cM apart were treated as an identical one [26]. Furthermore, QTL were named in accordance with the International rules of Genetic Nomenclature (http://wheat.pw.usda.gov/ggpages/wgc/98/Intro.htm). The ‘sicau’ represents Sichuan Agricultural University.

### Marker development and QTL validation

Two SNP markers were converted to Kompetitive allele-specific PCR (KASP) markers as previously described [26]. The KASP marker, *KASP-AX-111112626* (Table S3), tightly linked to *QKL.sicau-AM-3B*, was used to verify the effect of *QKL.sicau-AM-3B* in MP population. While *KASP-AX-108974756* (Table S3) was used to validate the effect of *QKW.sicau-AM-4B* in two populations (AAs and API) with different genetic backgrounds.

As F_3_ is a segregating generation, we selected 15 F_3_ kernels from each line of MP, AAs, and API populations for germination and grew them in greenhouse. Leaves of 15 seedlings were all collected and mixed for DNA extraction representing F_2_ genotype. High-quality genomic DNA was extracted using the Plant Genonic DNA Kit (Tiangen Biotech, Beijing, China), and was then used to do genotyping using KASP markers. Details of the amplification reaction system and conditions were listed in Table S3. The lines were divided into two groups (Data set 1 and 2) based on the genotyping results. Data set 1 represented lines with homozygous alleles from Ailanmai or LM001, whereas Data set 2 represented lines with homozygous alleles from the other parents. Lines with heterozygous genotype were not included for analysis. Finally, we evaluated the differences in KL or KW between the two groups with the independent sample *t*-test (*P* < 0.05) to determine the effects of the major QTL.

The flanking marker *AX-111112626* was included in CWL natural population genotyped using the Wheat 55 K SNP array and *AX-108974756* was included in the CWC natural population genotyped using Wheat 660 K SNP array*.* According to the genotype of these two flanking markers in the CWL and CWC populations. The lines were divided into two groups: (1) lines with identical genotype as Ailanmai. (2) lines with identical genotype as LM001. The BLUP values of KL and KW data from all environments of CWL and CWC were used to analyze the differences with the independent sample *t*-test (*P* < 0.05) between the two groups.

### Physical intervals of the major QTL and comparison with previously reported QTL

In order to predicate physical intervals of the major QTL identified in this study, the sequences of their flanking markers were used to blast against (*E*-value of 1e-5) genomes sequences of the wild emmer wheat ‘Zavitan’ WEWseq v2 (http://202.194.139.32/blast/blastresult.php) [27] and the International Wheat Genome Sequencing Consortium (IWGSC) Chinese Spring (CS) RefSeq v2.1 (https://urgi.versailles.inrae.fr/download/iwgsc/IWGSC_RefSeq_Assemblies/v2.1/) [28]. The annotations and functions of genes were retrieved on UniProt (http://www.uniprot.org/). We compared physical distances by anchoring flanking marker sequences of KL and KW QTL obtained in previous studies on CS to indicate whether the currently determined QTL were novel.

Furthermore, to identify the possible regulatory genes of KL and KW, the spatio-temporal expression patterns of the genes that were identified in the intervals of *QKL.sicau-AM-3B* and *QKW.sicau-AM-4B* were analyzed using the Triticeae Multi-omics Center website (https://202.194.139.32/expression/index.html). For *TraesCS4B03G0584900* and *TraesCS4B03G0585000*, their spatio-temporal expression patterns were analyzed using Chinese Spring cv-1 Development (single) on the Triticeae Multi-omics Center website (https://202.194.139.32/expression/index.html).

## Results

### Phenotypic data analyses

**Fig. 1:**
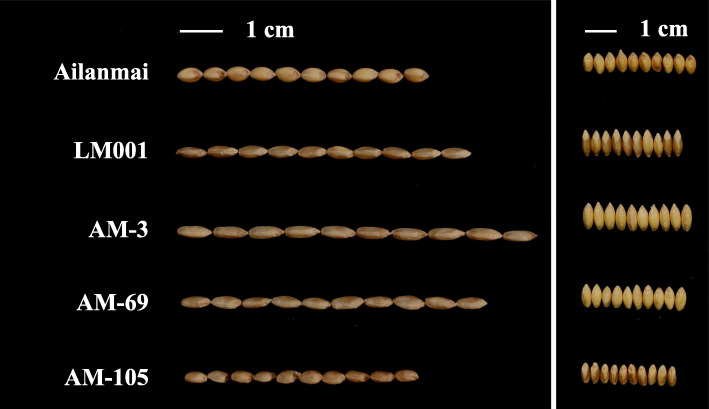
Phenotypes of the parents Ailanmai, LM001 and partial RILs. Comparison of kernel length and width among parents Ailanmai, LM001 and partial RIL (i.e. AM-3, AM-69 and AM-105). The white bar represents the scale = 1 cm

LM001 showed longer and narrower kernel than Ailanmai (Fig. 1; Table 1). The values of the KL and KW in each environment showed a continuous distribution (Fig. S1a, b). The KL and KW ranged from 5.86 to 9.43 mm and from 2.74 to 4.16 mm, respectively, in the AM RIL population (Table 1). The standard deviation (STD) of KL and KW ranged from 0.45 to 0.60 and from 0.11 to 0.24, respectively. *H*^*2*^ of KL and KW were 0.79 and 0.72, respectively (Table 1). The result indicated that both KL and KW had high repeatability over testing environments, suggesting KL and KW were mainly controlled by genetic factors.

**Table 1:** Phenotype variance explained (PVE), standard deviation (STD) and the broad-sense heritability (*H*^*2*^) of kernel length (KL) and width (KW) for the parents and AM RIL population in different environments

Trait	Environment	Parents		Ailanmai × LM001 (AM)			
		Ailanmai	LM001	Range	Mean	STD	*H*^*2*^
KL (mm)	2017CZ	N	N	5.96–9.06	8.11	0.56	0.79
	2018CZ	6.28^**^	8.15	5.90–9.00	8.14	0.58	
	2019CZ	6.67^**^	8.65	6.59–9.40	8.46	0.52	
	2020CZ	6.28^**^	8.35	6.51–9.43	8.48	0.54	
	2020WJ	6.56^**^	8.49	5.86–9.16	8.25	0.60	
	2020YA	6.70^**^	8.52	7.18–9.11	8.46	0.45	
	BLUP	6.58^**^	8.39	6.47–9.04	8.31	0.46	
KW (mm)	2017CZ	N	N	2.89–3.72	3.43	0.19	0.72
	2018CZ	3.90^**^	3.34	3.16–3.82	3.54	0.15	
	2019CZ	3.92^**^	3.42	3.21–4.16	3.64	0.20	
	2020CZ	3.96^**^	3.35	2.74–3.95	3.49	0.24	
	2020WJ	3.91^**^	3.33	3.19–4.14	3.58	0.18	
	2020YA	3.81^**^	3.37	2.92–3.87	3.46	0.20	
	BLUP	3.82^**^	3.38	3.26–3.80	3.52	0.11	

*KL* kernel length, *KW* kernel width, *CZ* Chongzhou, *WJ* Wenjiang, *YA* Ya’an, *STD* standard deviation, *H*^*2*^ the broad-sense heritability, *BLUP* phenotype values based on BLUP, *N* the data was missing; ^**^ Significance at the 0.01 probability level; ^*^ Significance at the 0.05 probability level

### Correlation analyses between kernel traits and other yield-related traits

Significant and positive correlations for KL and KW were detected in most different environments (*P* < 0.05). The correlation coefficients ranged from 0.62 to 0.82 for KL and from 0.29 to 0.45 for KW, respectively (Table 2).

**Table 2:** Correlation coefficients of KL and KW in different environments

Trait	Environment	2017CZ	2018CZ	2019CZ	2020CZ	2020WJ	2020YA
KL	2017CZ	1					
	2018CZ	0.72^**^	1				
	2019CZ	0.67^**^	0.82^**^	1			
	2020CZ	0.62^**^	0.70^**^	0.77^**^	1		
	2020WJ	0.65^**^	0.71^**^	0.81^**^	0.69^**^	1	
	2020YA	0.76^**^	0.79^**^	0.80^**^	0.72^**^	0.75^**^	1
KW	2017CZ	1					
	2018CZ	0.33^**^	1				
	2019CZ	0.45^**^	0.12	1			
	2020CZ	0.43^**^	0.29^**^	0.36^**^	1		
	2020WJ	0.26	0.33^**^	0.33^**^	0.45^**^	1	
	2020YA	0.43^**^	0.29^**^	0.36^**^	0.37^**^	0.29^**^	1

*KL* kernel length, *KW* kernel width, *CZ* Chongzhou, *WJ* Wenjiang, *YA* Ya’an; ^**^ Significance at the 0.01 probability level; ^*^ Significance at the 0.05 probability level

The BLUP datasets of kernel size and yield-related traits were employed to evaluate their relationships. Correlation analysis showed that significant correlations (*P* < 0.05) were observed between KL and SL, UIL, TKW, FLL (*r* = 0.32 to 0.66; Fig. S2a, c, d, and g). However, there were no significant correlations between KL and ETN, GNS, KW, FLW (*r* = − 0.061 to 0.10; Fig. S2b, e, f, and h). Moreover, KW showed significant correlations (*P* < 0.05) with TKW (*r* = 0.31; Fig. S2l), but the other six agronomic traits (SL, ETN, UIL, GNS, FLL and FLW) were not significantly correlated to the KW (*r* = − 0.16 to 0.13; Fig. S2i, j, k, m, n, and o).

### QTL detection

A total of eleven putative QTL associated with KL (six QTL) and KW (five QTL) were identified in the AM population and they were located on chromosomes 1B, 2A, 2B, 3B, 4B, 6A, 6B, and 7A (Table 3).

**Table 3:** Quantitative trait loci (QTL) mapping for kernel length (KL) and kernel width (KW)

Trait	QTL	Environment	Chromosome	Left Marker	Right Marker	LOD	PVE (%)	Add	CI
KL	*QKL.sicau-AM-6A*	2019CZ	6A	*AX-109843320*	*AX-108755285*	2.55	4.56	−0.12	10.5–21.5
	*QKL.sicau-AM-2B*	2018CZ	2B	*AX-108730087*	*AX-110447950*	3.06	5.38	−0.20	21.5–37.5
	*QKL.sicau-AM-3B*	2018CZ	3B	*AX-111112626*	*AX-110375013*	4.22	17.57	−0.24	1.5–7.5
		2019CZ		*AX-111112626*	*AX-110375013*	15.84	44.28	−0.35	4.5–7.5
		2020CZ		*AX-111112626*	*AX-110375013*	12.45	37.50	−0.35	3.5–7.5
		2020WJ		*AX-111112626*	*AX-110375013*	7.69	27.70	−0.32	2.5–7.5
		2020YA		*AX-111112626*	*AX-110375013*	10.03	32.55	−0.26	2.5–7.5
		BLUP		*AX-111112626*	*AX-110375013*	11.58	34.55	−0.29	2.5–7.5
	*QKL.sicau-AM-3B.1*	2017CZ	3B	*AX-108914590*	*AX-108727907*	3.90	13.57	−0.21	10.5–13.5
	*QKL.sicau-AM-4B*	BLUP	4B	*AX-109410422*	*AX-94486277*	3.62	15.68	0.03	81.5–83.0
	*QKL.sicau-AM-6B*	2017CZ	6B	*AX-109336882*	*AX-110062048*	3.37	18.59	0.19	127.5–130.5
KW	*QKW.sicau-AM-2A*	BLUP	2A	*AX-108949998*	*AX-109355803*	3.38	22.96	0.03	134.5–140.5
	*QKW.sicau-AM-7A*	BLUP	7A	*AX-108885515*	*AX-109302546*	2.86	26.22	−0.03	91.5–97.5
	*QKW.sicau-AM-1B*	BLUP	1B	*AX-111062860*	*AX-110067443*	4.98	29.08	0.04	79.5–81.5
	*QKW.sicau-AM-2B*	2019CZ	2B	*AX-109897880*	*AX-110598098*	2.96	29.82	0.06	11.5–13.5
	*QKW.sicau-AM-4B*	2017CZ	4B	*AX-108974756*	*AX-111451315*	7.77	25.17	0.10	12.5–14.5
		2018CZ		*AX-108974756*	*AX-111451315*	10.10	38.44	0.09	12.5–14.5
		2019CZ		*AX-108955591*	*AX-110915030*	3.81	13.91	0.07	14.5–15.5
		2020CZ		*AX-108955591*	*AX-110915030*	8.91	29.97	0.13	14.5–15.5
		2020WJ		*AX-108955591*	*AX-110915030*	5.98	23.31	0.08	14.5–15.5
		2020YA		*AX-108955591*	*AX-110915030*	5.58	24.02	0.09	14.5–15.5
		BLUP		*AX-108955591*	*AX-110915030*	18.17	39.01	0.08	14.5–15.5

*KL* kernel length, *KW* kernel width, *CZ* Chongzhou, *WJ* Wenjiang, *YA* Ya’an, *LOD* logarithmic odds, *PVE* phenotype variation values. Add: additive effect of a QTL, positive values indicate that alleles from Ailanmai are increasing the trait scores, and negative values indicate that alleles from LM001 are increasing the trait scores. CI: confidence interval of the QTL

Six QTL for KL explained 4.56 to 44.28% of the PVE. *QKL.sicau-AM-3B*, a major and stable locus, was detected in five environments and BLUP data, and explained 17.57 to 44.28% of the PVE. The positive allele was from LM001 (Table 3). The remaining five QTL detected in a single or two environments explained between 4.56 and 18.59% of the PVE.

Furthermore, five QTL for KW explained 13.91 to 39.01% of the PVE. *QKW.sicau-AM-4B*, a major QTL, detected in all the six environments and also the BLUP data. This locus could explain 13.91 to 39.01% of the PVE, and the positive allele was contributed by Ailanmai (Table 3). The other four QTL were detected in less than three environments, and they explained between 22.96 and 29.82% of the PVE (Table 3). Furthermore, twenty-eight QTL were detected using QE interaction analysis (Table S4). *QKL.sicau-AM-3B* controlling KL and *QKW.sicau-AM-4B* controlling KW were simultaneously identified by multi-environmental and individual environmental analyses, further showing that they were major and stable QTL.

### Verification of the major QTL in different genetic backgrounds

**Fig. 2:**
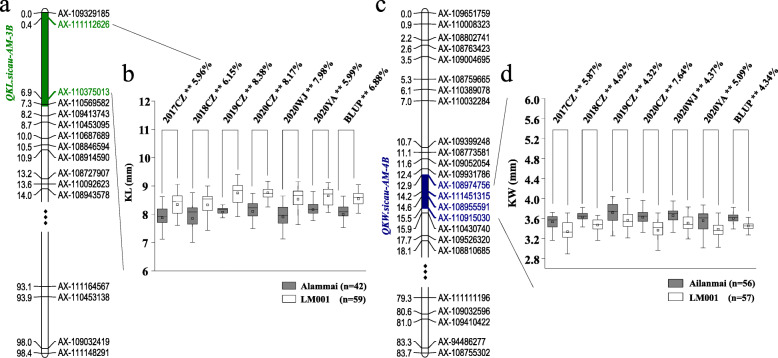
Major QTL *QKL.sicau-AM-3B* and *QKW.sicau-AM-4B* and their effects. (**a**): Genetic map of chromosome 3B; the green area was the interval of *QKL.sicau-AM-3B*; (**b**): effect of *QKL.sicau-AM-3B* shown as box plots calculated after grouping the AM population into two classes based on the flanking markers. Ailanmai and LM001 indicate the lines with and without positive alleles of *QKL.sicau-AM-3B*; (**c**): genetic map of chromosome 4B; The blue area was the interval of *QKW.sicau-AM-4B*; (**d**): effect of *QKW.sicau-AM-4B* shown as box plots calculated after grouping the AM population into two classes based on the flanking markers. CZ: Chongzhou; WJ: Wenjiang; YA: Ya’an; ^**^ significance at the 0.01 probability level, ^*^ significance at the 0.05 probability level. Differences between the two groups were labeled below the environmental names and BLUP

A KASP marker, *KASP-AX-111112626*, tightly linked to *QKL.sicau-AM-3B* and one, *KASP-AX-108974756*, tightly linked to *QKW.sicau-AM-4B* were developed to validate their effects in different genetic backgrounds (Table S3; Fig. 2).

**Fig. 3:**
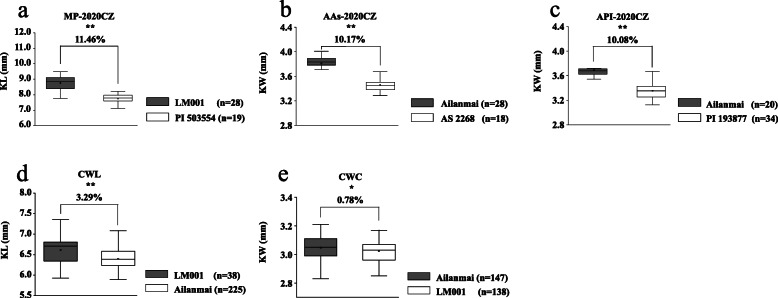
Verification of *QKL.sicau-AM-3B* and *QKW.sicau-AM-4B* in two and three different genetic background populations, respectively. (**a**), (**d**): Effects of *QKL.sicau-AM-3B* in the MP and the CWL validation populations, respectively; (**b**), (**c**), and (**e**): effects of *QKW.sicau-AM-4B* in the three populations (i.e. AAs, API and CWC). CZ: Chongzhou; ^**^ significance at the 0.01 probability level, ^*^ significance at the 0.05 probability level

According to the polymorphism of *KASP-AX-111112626*, the lines were divided into two groups in the AM RIL population: lines with Ailanmai homozygous allele and lines with LM001 homozygous allele (excluding heterozygosis). The group with positive allele of *QKL.sicau-AM-3B* (from LM001) had significantly greater values than that with negative one (from Ailanmai) in each environment and BLUP data set (*P* < 0.05; Fig. 2a and b). Likewise, the lines from CWL population were divided into two groups. The group with positive allele of *QKL.sicau-AM-3B* had 3.29% higher values than that with negative one (*P* < 0.05; Fig. 3d). In MP population, the lines with positive allele had 11.46% higher values than those with negative one, indicating that *QKL.sicau-AM-3B* is indeed a major QTL controlling KL (Fig. 3a).

Additionally, according to the polymorphism of *KASP-AX-108974756*, the lines from AM population were also divided into two groups. The group with positive allele of *QKW.sicau-AM-4B* had significantly higher values than that with negative one in six environments and BLUP data set (*P* < 0.05; Fig. 2c, d). In CWC population, the group with positive allele of *QKW.sicau-AM-4B* had significantly 0.78% greater values than that with negative one (*P* < 0.05; Fig. 3e). In AAs and API populations, the group with positive allele from Ailanmai had significantly greater values than that without this allele, and the differences between the two groups were 10.17 and 10.08%, respectively, with an average of 10.13% in two validation populations, indicating that *QKW.sicau-AM-4B* is also a major QTL controlling KW (Fig. 3b, c).

### Effects of *QKL.Sicau-AM-3B* and *QKW.Sicau-AM-4B* on TKW in AM population

In AM population, the positive alleles of *QKL.sicau-AM-3B* and *QKW.sicau-AM-4B* were from LM001 and Ailanmai, respectively (Table 3). The effects of *QKL.sicau-AM-3B* and *QKW.sicau-AM-4B* on TKW were further analyzed (Fig. 5). Compared with those without any of the alleles increasing KL and KW, lines possessed the positive allele of *QKL.sicau-AM-3B* but not that of *QKW.sicau-AM-4B* increased TKW by 1.65%; lines possessed that of *QKW.sicau-AM-4B* but not that of *QKL.sicau-AM-3B* increased TKW by 1.49%; and those with the combination of positive alleles of both *QKL.sicau-AM-3B* and *QKW.sicau-AM-4B* significantly increased TKW by up to 6.01% (*P* < 0.01). Besides, lines with the combination of positive alleles of *QKL.sicau-AM-3B* and *QKW.sicau-AM-4B* significantly increased TKW by 4.29 and 4.46% (*P* < 0.05), respectively, compared to those with the positive allele of the *QKL.sicau-AM-3B* only or the *QKW.sicau-AM-4B* only (Fig. 5). In addition, the binary linear regression analysis on TKW showed that the path coefficient of KL was 0.43, and that of KW was 0.02, indicating that KL contributes more to TKW than KW (Table S5).

## Discussion

### Relationship between kernel size and other agronomic traits

In this study, we evaluated the correlation coefficients between kernel size and other agronomic traits (Fig. S2). Positive and significant correlations were observed between KL, KW, and TKW (*P* < 0.05; Fig. S2d, l). The result indicated that the selection of larger kernels might lead to indirect selection of heavier kernels [29]. Kernel size, like KL and KW, greatly influences TKW. For example, Cui et al. found that compared with other kernel traits, KW has the largest effect on TKW [30]. Liu et al. also reported that TKW was mainly affected by KW [31]. In the current study, KL likely contributed more to TKW than KW (Fig. 5; Table S5), suggesting that increasing KL through utilization of positive allele of *QKL.sicau-AM-3B* may be more effective in increasing TKW than KW contributed by positive allele of *QKW.sicau-AM-4B* at tetraploid level. As expected, KL and KW were positively correlated with UIL (Fig. S2c, k). A longer UIL contributed to ventilation, light transmittance, and lower relative humidity of spikes, thus reducing the possibility of occurrence of diseases and insect pests such as scab, which was conducive to dry matter accumulation and affects kernel size [32]. KL and FLL showed significant positive correlation, and KW was positively correlated with FLW (*P* < 0.05; Fig. S2g, o). Theoretically, FLL and FLW determined the flag leaf area that was proportional to whether it had a strong assimilation tissue, vascular bundle area and these factors determined the kernel filling intensity of wheat, which was closely correlated with kernel size [33]. Furthermore, the results indicated that larger flag leaves increased yield by providing more photosynthetic nutrient to kernel [34]. The above conclusions provided a scientific basis for evaluating complex relationships among wheat yield components, which will be helpful in understanding increase of wheat yield.

### Stable and novel QTL controlling KL and KW

We compared the major QTL identified in this study with those detected in previous studies through aligning physical positions of their closest markers (Table S6).

**Fig. 4:**
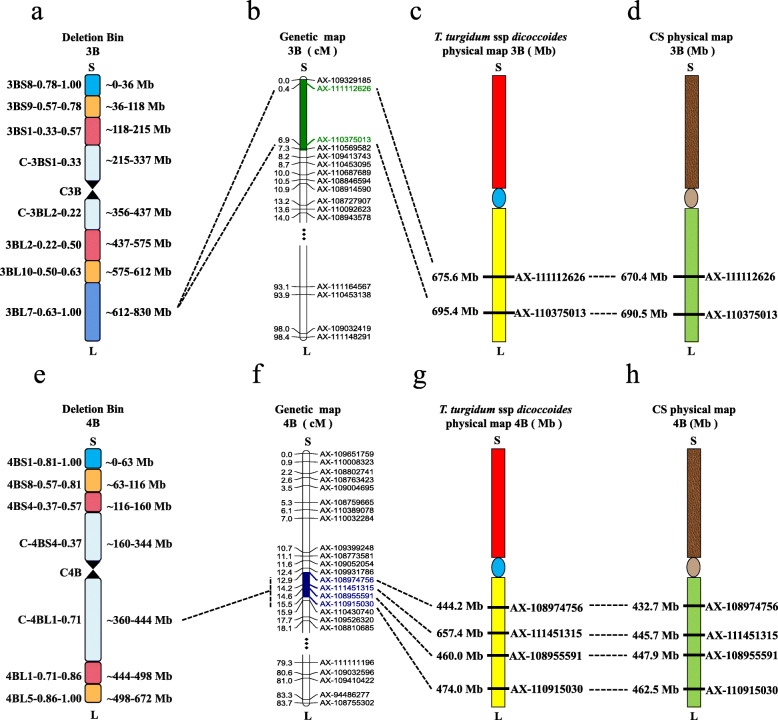
The physical interval of *QKL.sicau-AM-3B* and *QKW.sicau-AM-4B*. (**a**): Deletion bin map of wild emmer chromosome 3B; (**b**) partial genetic map of wheat chromosome 3B; (**c**): physical map of *T. turgidum ssp. dicoccoides* chromosome 3B; (**d**): physical map of CS chromosome 3B; (**e**): deletion bin map of wild emmer chromosome 4B; (**f**): partial genetic map of wheat (*Triticum aestivum* L.) chromosome 4B; (**g**): physical map of the wild emmer chromosome 4B; (**h**): physical map of CS chromosome 4B. The green and blue areas are the intervals of *QKL.sicau-AM-3B* and *QKW.sicau-AM-4B*, respectively, and the central area is centromere

*QKL.sicau-AM-3B* was located between 675.6 and 695.4 Mb in the deletion bin 3BL7–0.63-1.00 on chromosome arm 3BL in wild emmer (Fig. 4a, c), which was different from previously reported KL-related QTL (Table S6). For example, *QGl-3B.1* was detected on chromosome arm 3BS at 52.1–53.2 Mb [35]. And *QGl.ccsu-3B.1* was flanked by marker *Xgwm376* (38.9 Mb) [36]. Two QTL, *QKL.ndsu.3B* and *QKL.ndsu.3B.1*, were located at 211.7–216.3 Mb and 233.9–244.6 Mb, respectively [37]. And *qKL.3B* was identified on chromosome arm 3BS with the closest marker *Xgwm429* (20.5 Mb) [38]. Thus, these results indicated *QKL.sicau-AM-3B* may be a novel QTL controlling KL detected in the present study.

For KW, *QKW.sicau-AM-4B* was located between 444.2 and 474.0 Mb in the deletion bin 4BL1–0.71 and 4BL1–0.71-0.86 on chromosome arm 4BL in wild emmer (Fig. 4e, g). Comparison of physical positions of *QKW.sicau-AM-4B* with those reported previously suggested that they were not overlapped (Table S6). For example, there were five QTL, *QKw.ncl-4B.1* [39], *QGw-4B.1* [35], *Size width 2011* [14], *QKw4B.1–7* [40], *QKw-4B.2* [41], and *kw-WY-4B-1.2* [30], associated with KW being detected on chromosome arm 4BS. *qKW-4B* was identified on chromosome arm 4BL with the closest marker *Xwmc657* (610.5 Mb) [38], and *qKW4B-1* was detected on chromosome arm 4BL at 610.1–649.1 Mb [8]. These results showed that *QKW.sicau-AM-4B* is probably a novel QTL controlling KW in wheat.

### Predictive genes in the intervals where major QTL were located

*QKL.sicau-AM-3B* was located between 675.6 and 695.4 Mb on wild emmer 3BL and between 670.4 and 690.5 Mb on CS 3BL by anchoring flanking markers *AX-111112626* and *AX-110375013* of *QKL.sicau-AM-3B* (Fig. 4a, b, c, and d). There were twenty-nine shared predicated genes (Table S7). Expression analyses showed that twenty-four genes can be expressed in kernel (Fig. S3a). Similarly, for *QKW.sicau-AM-4B*, it was mapped between 444.2 and 474.0 Mb on chromosome arm 4BL of wild emmer and 432.7 and 462.5 Mb on chromosome arm 4BL of CS by anchoring its flanking markers *AX-108974756* and *AX-110915030* (Fig. 4e, f, g and h). There were forty shared predicated genes (Table S7). Expression analyses showed that thirty-four genes can be expressed in kernel (Fig. S3b).

Of these sixty-nine genes, four genes were involved in kernel development. For example, *TRIDC3BG062390* encoded fructose-bisphosphate aldolase (FBA) and it had a higher expression in kernel than other genes (Fig. S3a). FBA is an important isozyme involved in plant metabolism, and it is directly involved in the fixation and distribution of photosynthate [42]. Cytosolic and plastidic FBAs were expressed in plant photosynthetic tissues [43]. FBA regulates kernel size development through affecting plant photosynthesis. In addition, there were two un-functional and annotated genes of wild emmer, *TRIDC4BG037810* and *TRIDC4BG037830*. Nonetheless, they were highly expressed in kernel at different growth stages (Fig. S4a, b). Therefore, we identified annotations of their orthologs in CS [28]. *TraesCS4B03G0584900* (*TRIDC4BG037810*) and *TraesCS4B03G0585000* (*TRIDC4BG037830*) encoded Heat-shock protein (HSP; Table S7) and were also highly expressed in kernel (Fig. S4c, d). HSP was widely reported in graminaceous plant [44]. At high temperature, the role of HSP is to ensure the normal growth of kernel in wheat through providing protection to soluble starch synthase [45]. It was reported that HSP, as a molecular chaperone, aids in refolding soluble starch synthases denatured by heat and thus prevents them from aggregating, which was beneficial to starch synthesis of in kernel [46]. Thus, these genes related to kernel development may provide information for fine mapping and gene cloning of these identified major and novel QTL.

### Utilization of elite alleles for kernel size from wheat related species

**Fig. 5:**
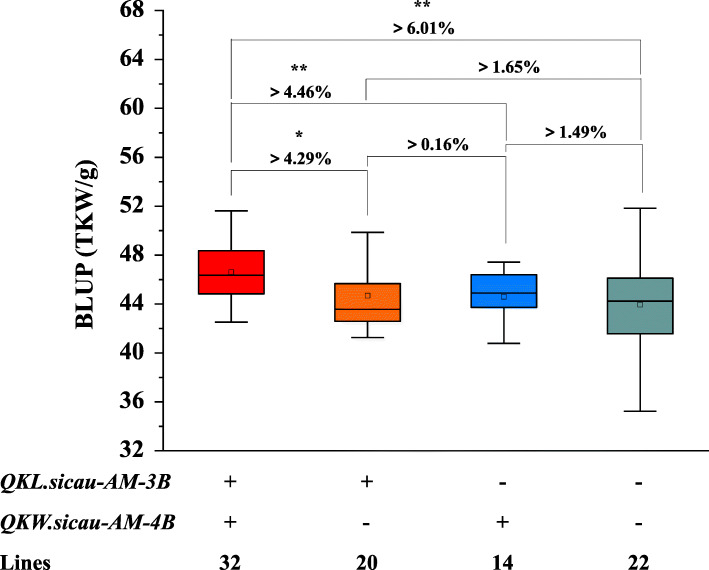
The effects of different combinations of *QKL.sicau-AM-3B* and *QKW.sicau-AM-4B* on increasing 1000-kernel weight (TKW) in the AM population. ‘+’ and ‘-’ represent lines with and without the positive allele of the corresponding QTL based on the genotype of flanking markers, respectively. ^**^ Significance at the 0.01 probability level, ^*^ significance at the 0.05 probability level

In the current study, two major, stably expressed, and novel QTL, *QKL.sicau-AM-3B* and *QKW.sicau-AM-4B* for kernel-related traits were identified from a wild emmer accession and a local landrace and validated in five populations with different genetic backgrounds. The combination of *QKL.sicau-AM-3B* and *QKW.sicau-AM-4B* had the largest additive effect on TKW (Fig. 5). These results suggest that they have a great potential in wheat breeding. Previous studies showed that pyramiding of choiceness genes was an effective method to improve a given trait [47]. In this study, we found some transgressive segregations in AM RIL. For example, AM-3 has longer and wider kernels than both parents (Fig. 1). Interestingly, AM-3 carries the positive alleles of both *QKL.sicau-AM-3B* and *QKW.sicau-AM-4B*, implying the possibility of pyramiding these two positive alleles from wheat related species in wheat breeding.

## Conclusions

Two major and novel QTL, *QKL.sicau-AM-3B* and *QKW.sicau-AM-4B*, were identified in AM RIL population. Both of them were successfully verified in their corresponding validation populations with newly developed KASP makers. Some genes involved in regulation of kernel growth and development were detected in the intervals where major KL and KW QTL were located. Significant correlations between kernel size and other agronomic traits were detected and discussed. KASP markers tightly linked the two major QTL could contribute greatly to subsequent fine mapping. This study indicated that wheat related species have great potentials for wheat yield improvement.

## Supplementary Information

**Additional file 1: Table S1**. **Information of CWL and CWC natural populations assessed in this study.**

**Additional file 2: Table S2**. **Environmental information of examined agronomic traits.**

**Additional file 3: Table S3.** Details of KASP primers used and the amplification reaction system and conditions in this study.

**Additional file 4: Figure S1.** Phenotypic distribution of kernel length (KL) and width (KW) at different environments and BLUP. (**a**): Frequency distribution map of KL; (**b**): frequency distribution map of KW. Black and gray arrows represent the parents Ailanmai and LM001, respectively.

**Additional file 5: Figure S2.** Correlation analysis for kernel traits (KL and KW) with (**a**) and (**i**): spike length (SL); (**b**) and (**j**): effective tiller number (ETN); (**c**) and (**k**): length of uppermost internode (UIL); (**d**) and (**l**): 1,000-kernel weight (TKW); (**e**) and (**m**): grain number per spike (GNS); (**f**): kernel width and length (KW & KL); (**g**) and (**n**): flag leaf length (FLL); (**h**) and (**o**): flag leaf width (FLW), respectively. ^**^ Significance at the 0.01 probability level, ^*^ significance at the 0.05 probability level.

**Additional file 6: Table S4.** Quantitative trait loci (QTL) detected in the QTL × environment interaction module (QE).

**Additional file 7: Table S5.** Results of the linear regression analysis on TKW.

**Additional file 8: Table S6.** Comparison of *QKL.sicau-AM-3B* and *QKW.sicau-AM-4B* with previous reported quantitative trait loci (QTL) or marker-trait associations (MTAs) for kernel length (KL) and kernel width (KW), respectively.

**Additional file 9: Table S7.** Predicated genes in the interval of *QKL.sicau-AM-3B* and *QKW.sicau-AM-4B*.

**Additional file 10: Figure S3.** Expression analysis of predictive genes in the interval of *QKL.sicau-AM-3B* (**a**) and *QKW.sicau-AM-4B* (**b**) in kernel.

**Additional file 11: Figure S4.** The expression of *TRIDC4BG037810* (**a**) and *TRIDC4BG037830* (**b**), and *TraesCS4B03G0584900* (**c**) and *TraesCS4B03G0585000* (**d**) in different growth stages of wild emmer and CS, respectively.

## Data Availability

The data that support the findings of this study are available in ‘figshare’ with the identifiers data DOIs, including dataset 1 (data of KL and KW of AM RILs in six environments, 10.6084/m9.figshare.14813094.v1), dataset 2 (the phenotypes and genotypes of MP, AAs and API populations, 10.6084/m9.figshare.14813109.v2). Remaining data generated or analyzed during this study are included in this published article and its Additional files.

## Abbreviations

QTL: Quantitative trait loci
KL: Kernel length
KW: Kernel width
TKW: 1000-kernel weight
SL: Spike length
ETN: Effective tiller number
UIL: Length of uppermost internode
GNS: Grain number per spike
FLL: Flag leaf length
FLW: Flag leaf width
AM: Ailanmai × LM001
RILs: Recombinant inbred lines
PVE: Phenotypic variance explained
KASP: Kompetitive allele-specific PCR
SNP: Single-nucleotide polymorphism
MP: LM001 × PI 503554
AAs: Ailanmai × AS 2268
API: Ailanmai × PI 193877
NPGS: The U.S. National Plant Germplasm System
CWL: Chinese wheat landraces
CWC: Chinese wheat cultivars
CZ: Chongzhou
WJ: Wenjiang
YA: Ya’an
BLUP: Best linear unbiased prediction
*H*^*2*^: Broad-sense heritability
LOD: Logarithm of odds
QE: QTL × Environment
CS: Chinese Spring
STD: Standard deviation
FBA: Fructose-bisphosphate aldolase
HSP: Heat-shock protein
