# Supplementary figures and images for: Genetic identification and characterization of chromosomal regions for kernel length and width increase from tetraploid wheat
Source: BMC Genomics. 2021 Sep 30;22:706. doi: 10.1186/s12864-021-08024-z (PMC8482559; doi:10.1186/s12864-021-08024-z)

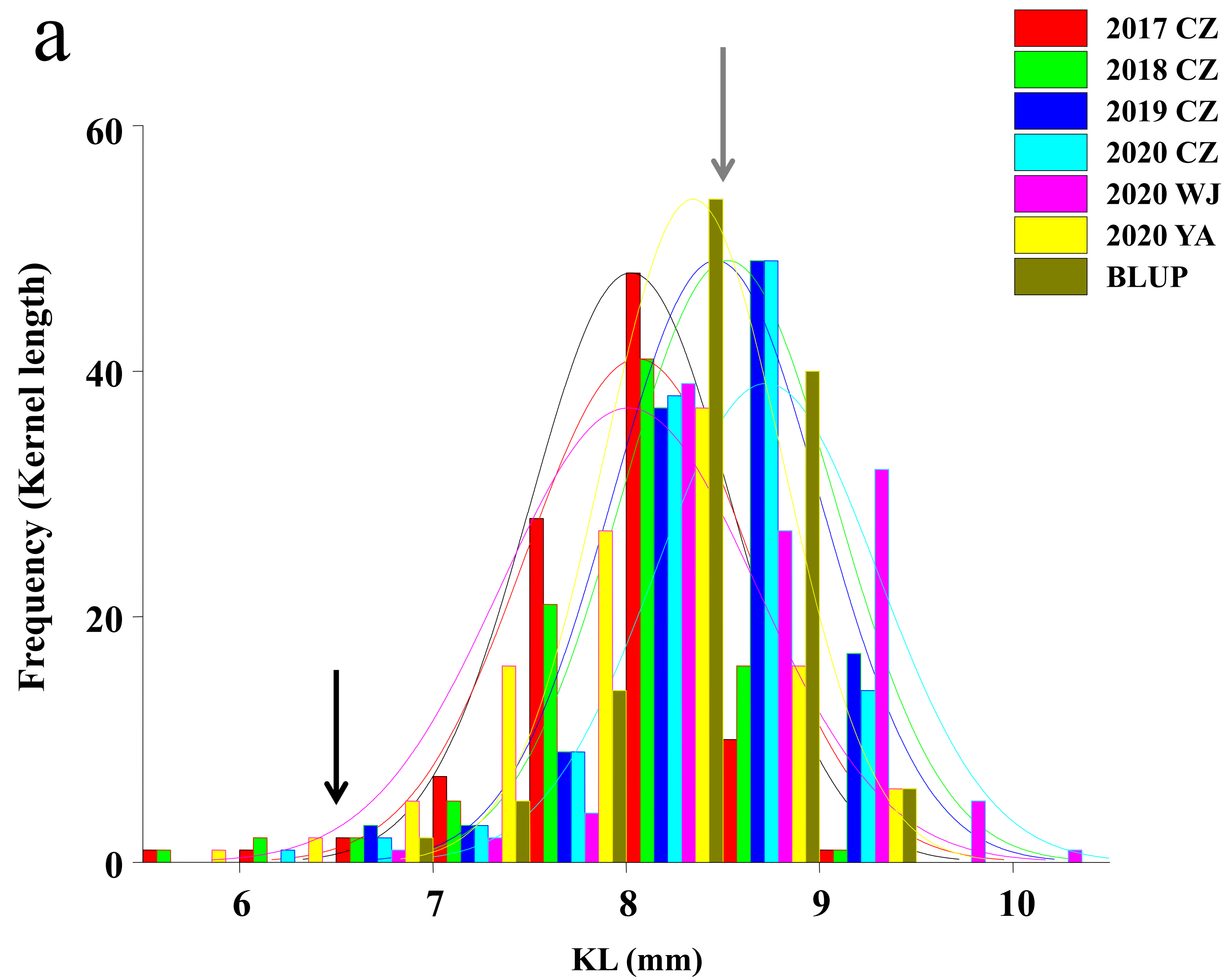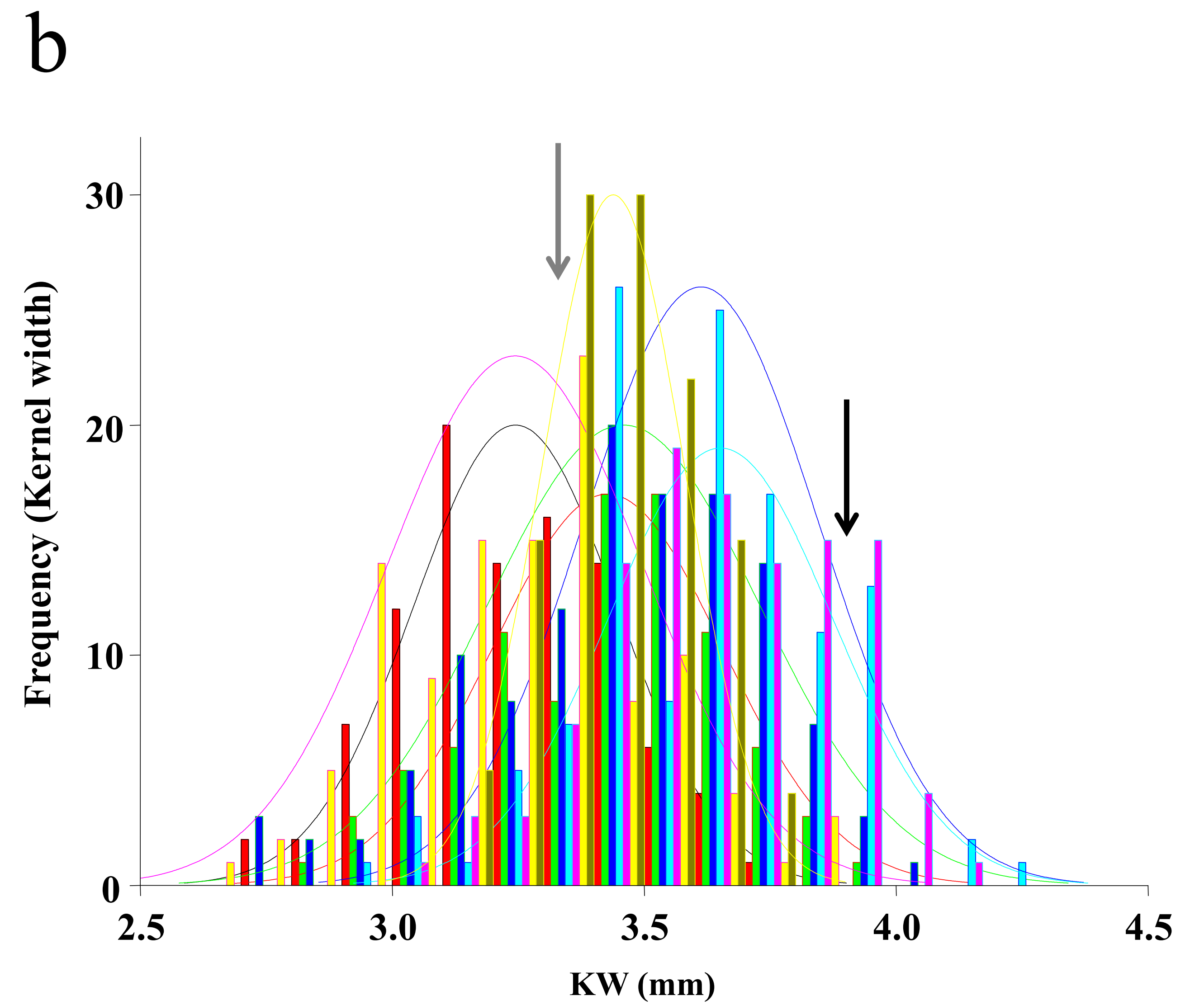

Supplement: Supplementary file 4 — Additional file 4: Figure S1. Phenotypic distribution of kernel length (KL) and width (KW) at different environments and BLUP. (a): Frequency distribution map of KL; (b): frequency distribution map of KW. Black and gray arrows represent the parents Ailanmai and LM001, respectively. [file 12864_2021_8024_MOESM4_ESM.pdf]

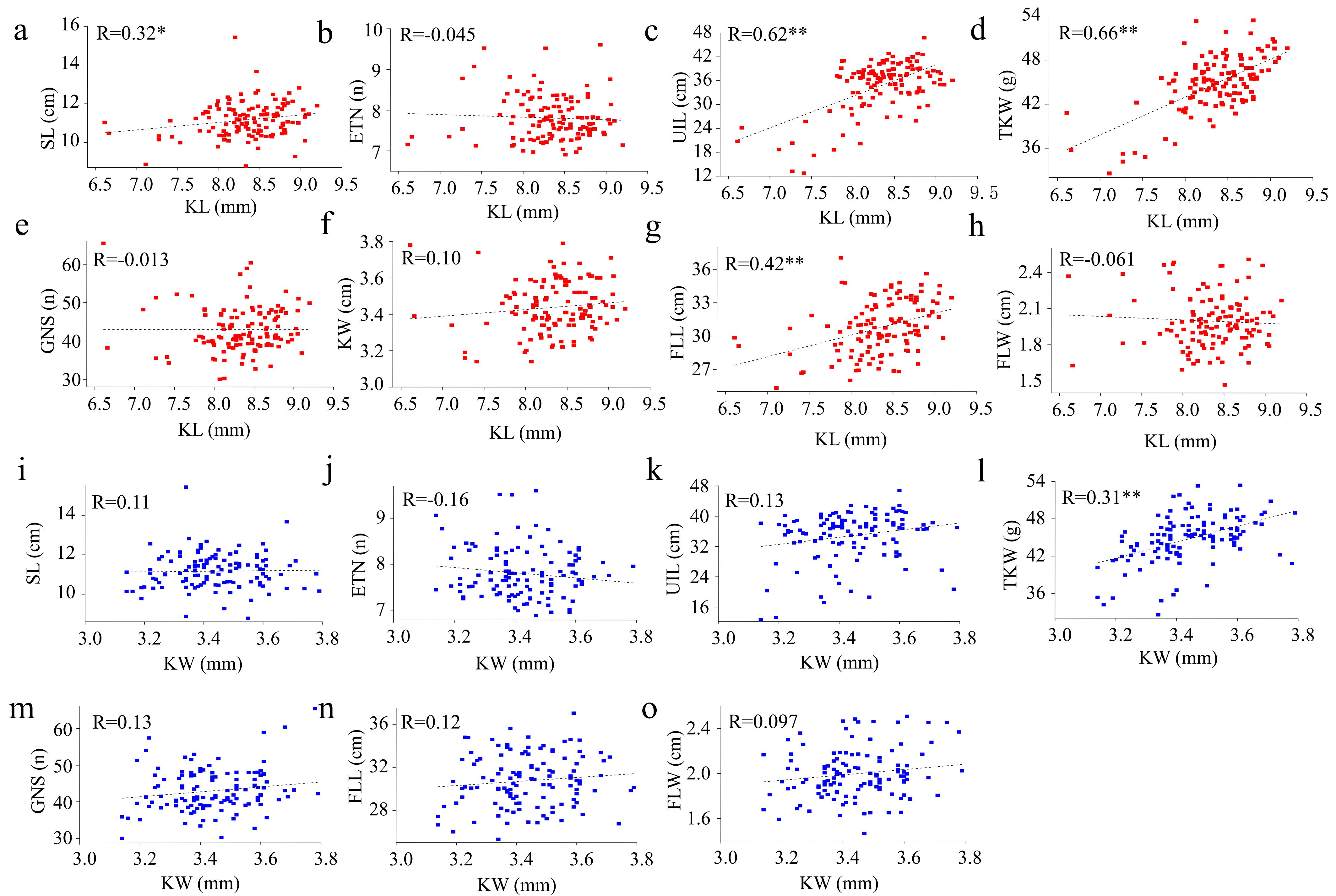

Supplement: Supplementary file 5 — Additional file 5: Figure S2. Correlation analysis for kernel traits (KL and KW) with (a) and (i): spike length (SL); (b) and (j): effective tiller number (ETN); (c) and (k): length of uppermost internode (UIL); (d) and (l): 1,000-kernel weight (TKW); (e) and (m): grain number per spike (GNS); (f): kernel width and length (KW & KL); (g) and (n): flag leaf length (FLL); (h) and (o): flag leaf width (FLW), respectively. ** Significance at the 0.01 probability level, * significance at the 0.05 probability level. [file 12864_2021_8024_MOESM5_ESM.pdf]

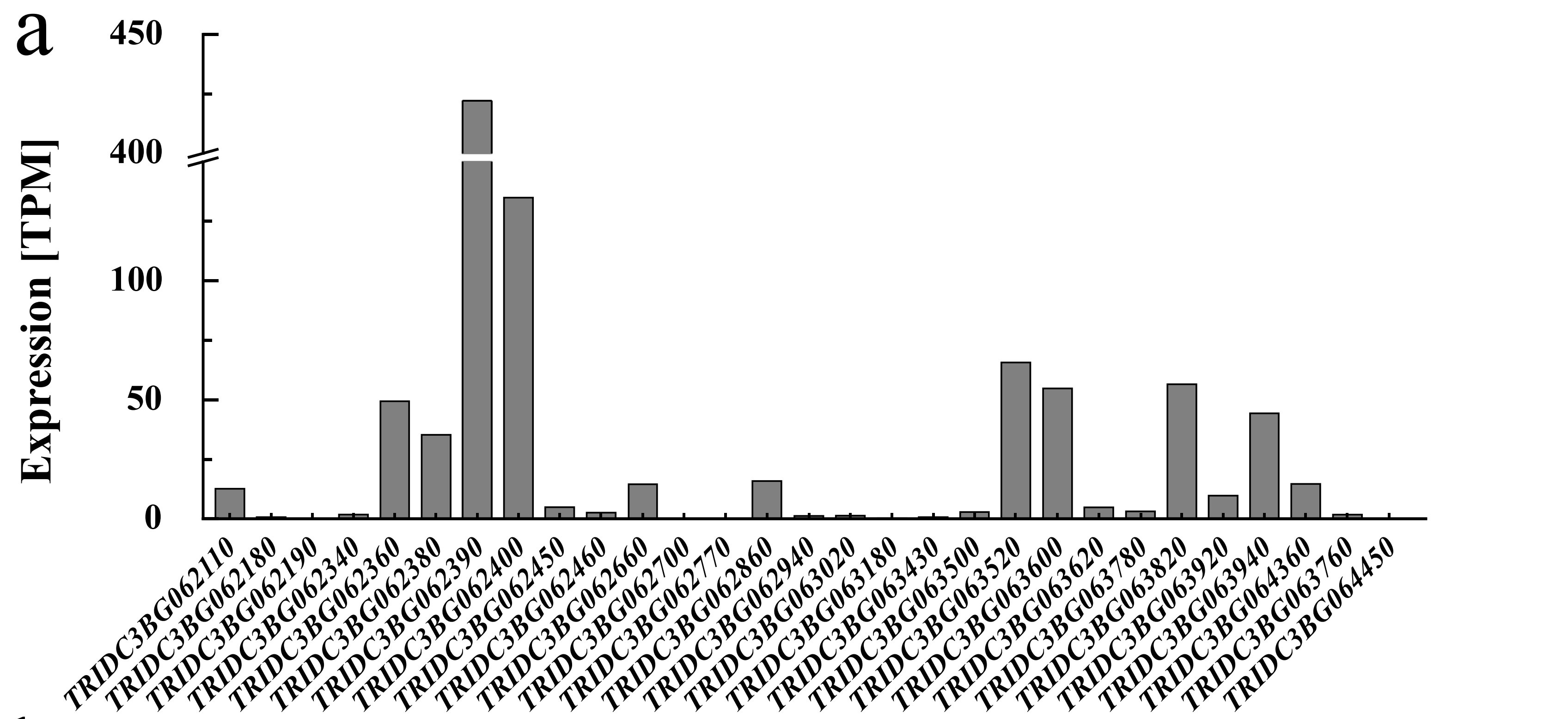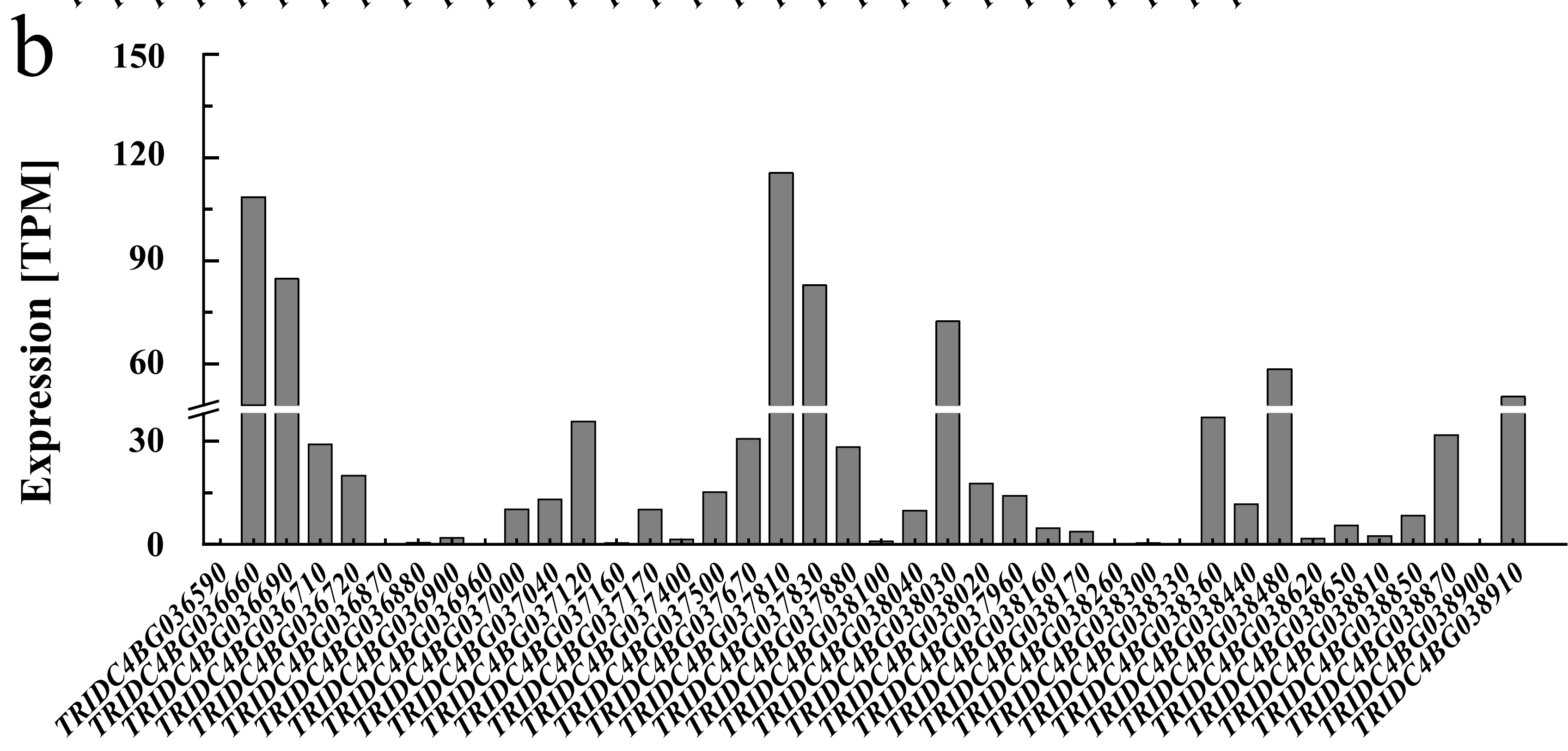

Supplement: Supplementary file 10 — Additional file 10: Figure S3. Expression analysis of predictive genes in the interval of QKL.sicau-AM-3B (a) and QKW.sicau-AM-4B (b) in kernel. [file 12864_2021_8024_MOESM10_ESM.pdf]

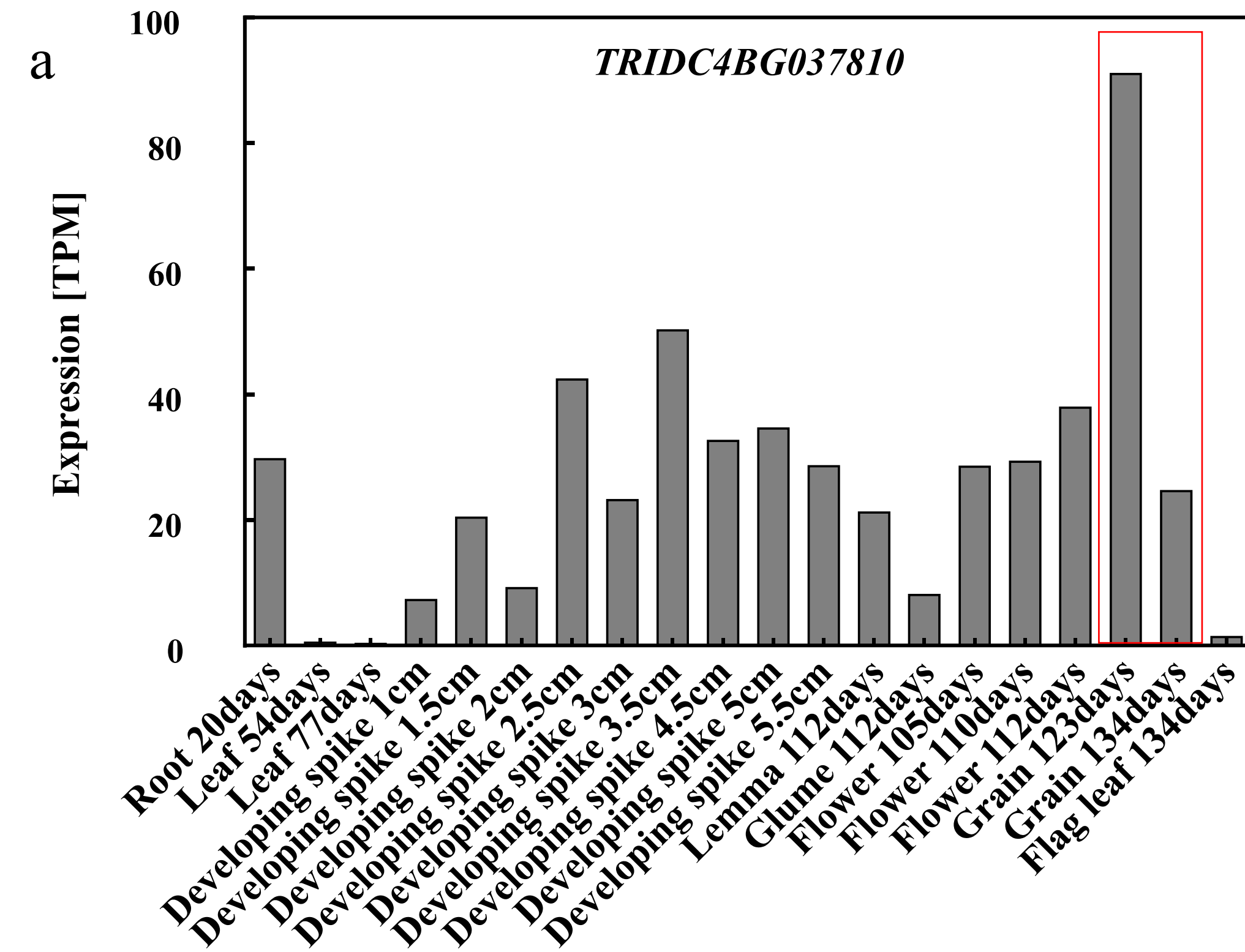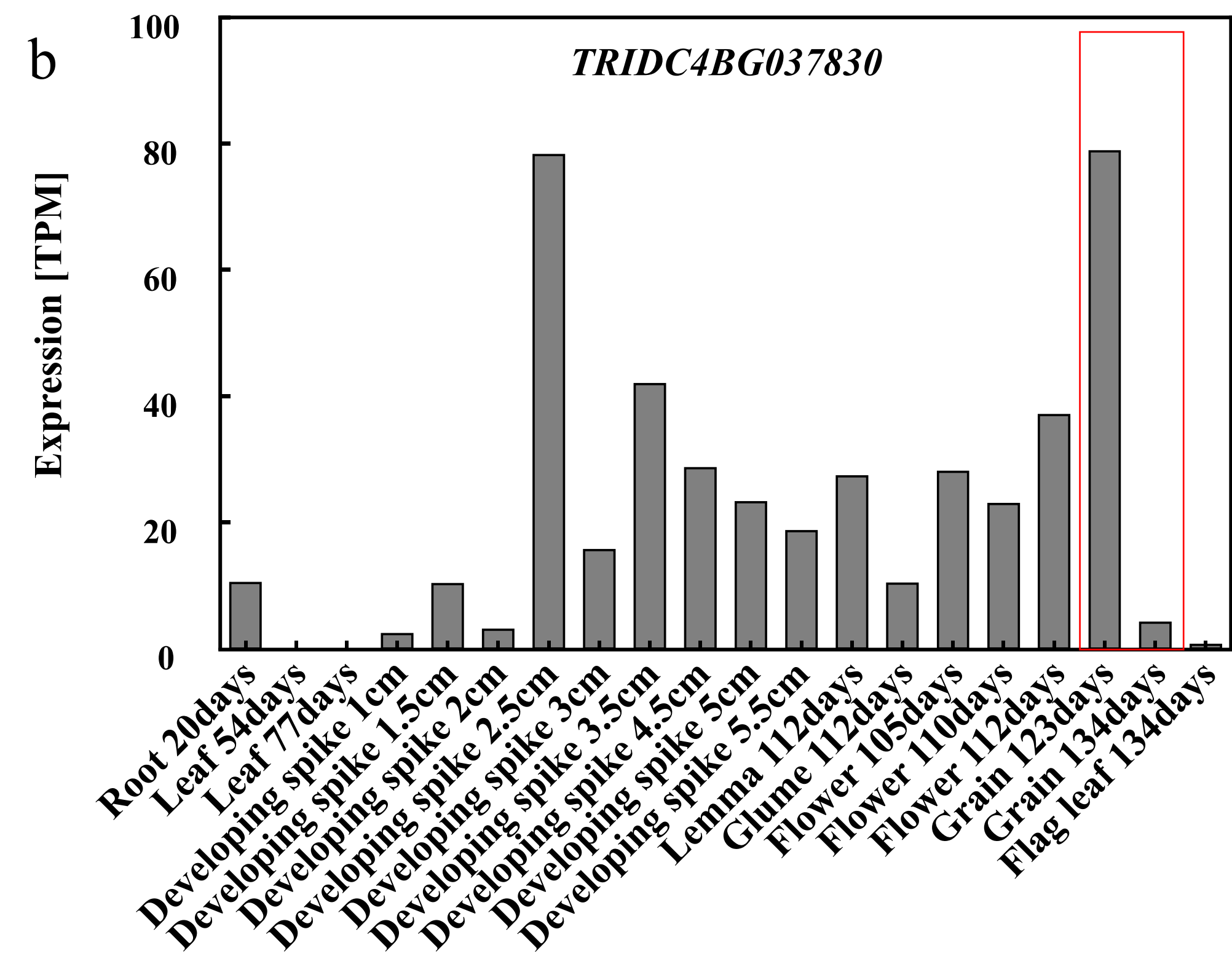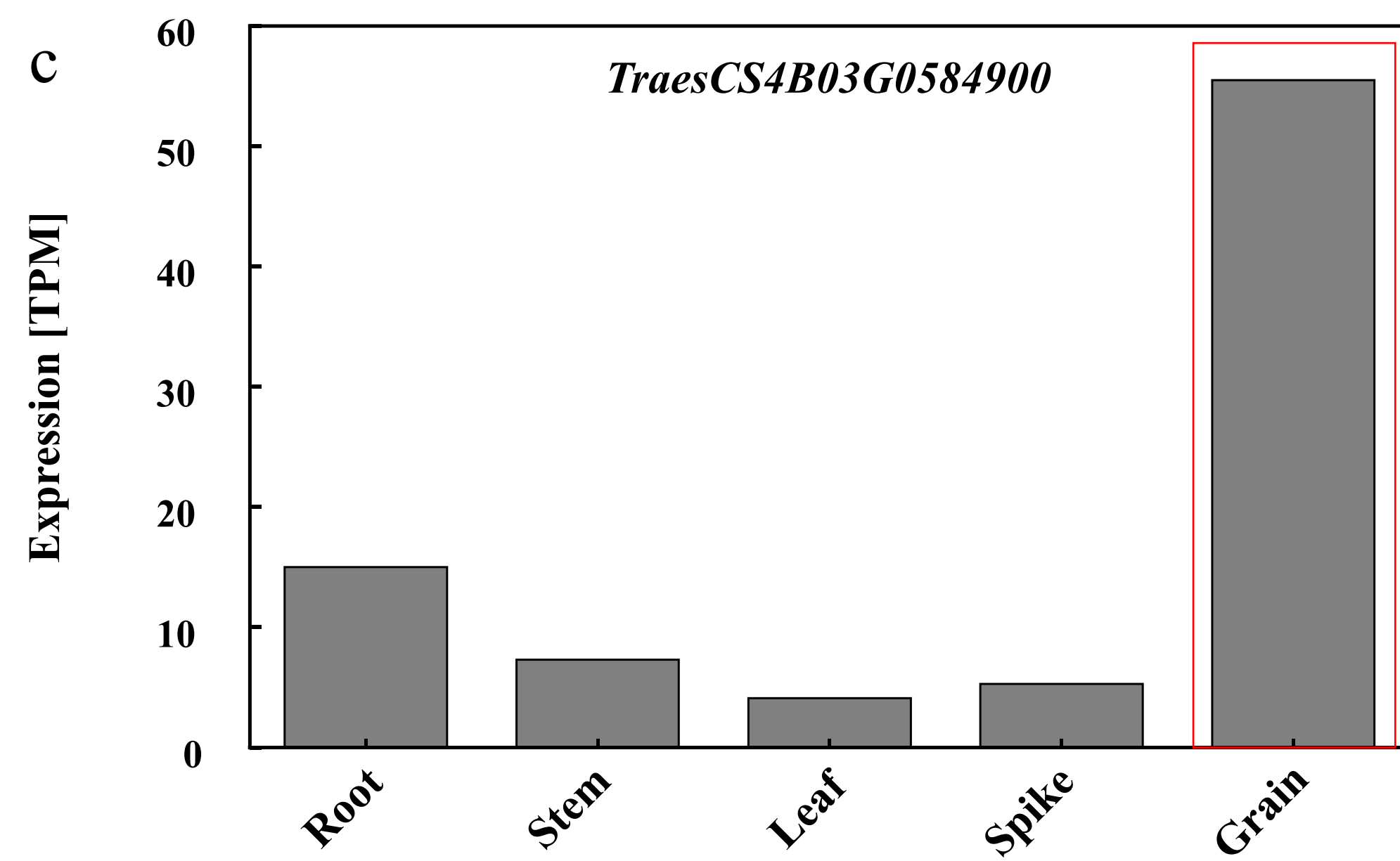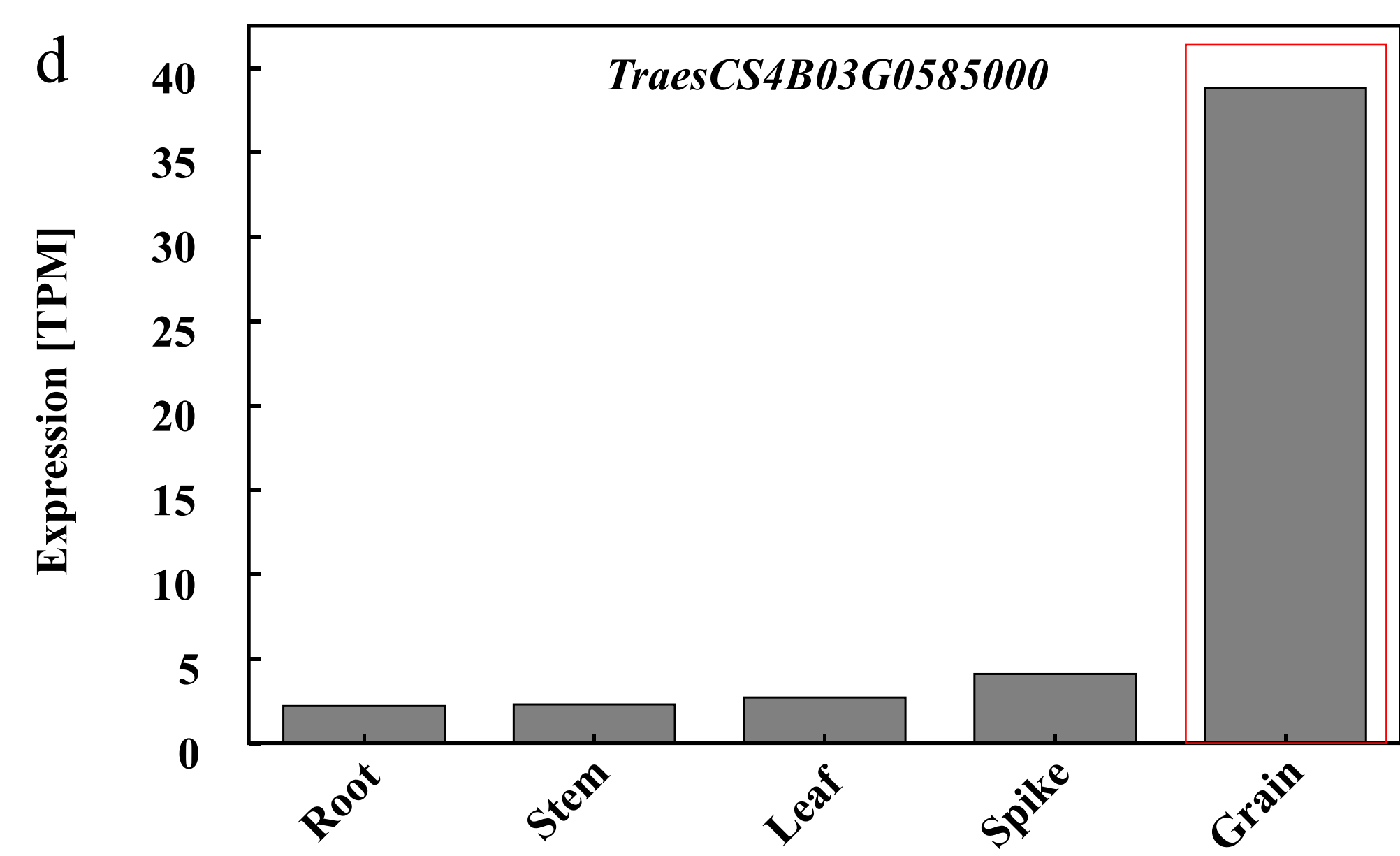

Supplement: Supplementary file 11 — Additional file 11: Figure S4. The expression of TRIDC4BG037810 (a) and TRIDC4BG037830 (b), and TraesCS4B03G0584900 (c) and TraesCS4B03G0585000 (d) in different growth stages of wild emmer and CS, respectively. [file 12864_2021_8024_MOESM11_ESM.pdf]
